# Challenges and prospects of polymeric biomaterials in immune engineering: A review

**DOI:** 10.1177/08853282261418177

**Published:** 2026-01-19

**Authors:** Boyka Andonova Lilova, Desislav Dinev, Probal Basu

**Affiliations:** 1 Department of Agrobiotechnology, Agro-bioinstitute, Sofia, Bulgaria; 2 Research and Development Innovation Consortium, Sofia Tech Park, Sofia, Bulgaria; 3 Faculty of Medicine, Medical University of Sofia, Sofia, Bulgaria; 4 Department of Biomaterials, Institute of Clinical Dentistry, University of Oslo, Oslo, Norway (Old Affiliation)

**Keywords:** polymers, biomaterials, macrophage, immune engineering

## Abstract

Immune engineering is a vast and rapidly developing field with a strong focus on polymeric materials. The importance of these biomaterials in immune engineering revolves around their ability to provide bioactive molecules to the target site, which influences the immune system. Their functional performance depends on various structural and functional properties of the biomaterial, which are also influenced by the host immune microenvironment. This work focuses on the current state of the art, the attributes of polymer-based biomaterials, and their limitations in immune engineering applications. This review not only elaborates on the advantages of polymeric biomaterials in immune engineering but also critically analyses the potential of these biomaterials in this field. The current work begins with the identification of key characteristics of polymer-based biomaterials for immune engineering, then explores different aspects of various polymeric materials and their importance in immune engineering applications. One of the key advantages of polymeric materials is that they can be efficiently designed to deliver bioactive molecules that significantly influence the host immune system. On the other hand, a notable limitation of these materials involves the development of adverse immune responses that can often occur due to the incompatibility of polymeric biomaterials with the host immune system. Finally, the review delves into the future perspectives and potential of these materials in immune engineering based personalized medicine and/or engineering living material (ELM) specific applications.

## Introduction

**Figure 1.**
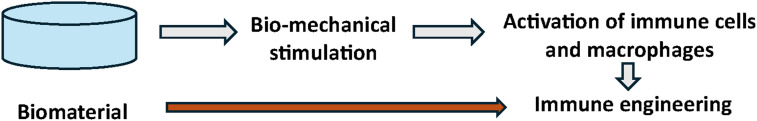
The essence of biomaterial-based approach for immune engineering.

Traditionally, polymeric biomaterials have been extensively utilised in a variety of biomedical applications as injectable or implantable tissue regeneration devices. However, these polymeric materials have been found to be promising for influencing the host immune system.^1^ Immune engineering is a vast and rapidly developing field with a strong focus on polymeric materials that provide not only structural support and integrity but also influence the immune system of the patient.^2^ Immune engineering methods utilise polymeric biomaterials that mimic the complexity of the immune system and facilitate a variety of immunological processes, such as antigen presentation, activating effector molecules, and T-cells through intricate interactions.^3^ The importance of polymer-based biomaterials in immune engineering applications revolves around their ability to provide bioactive molecules to the target site, which influences the immune system. Various polymer-based materials impact macrophage polarization, which facilitates immune engineering.^4^ These biomaterials exhibit important physicochemical characteristics such as mechanical strength, surface topography, roughness, wettability, and pore size, which can be tailored to meet specific immune tissue engineering needs through the activation of macrophages. Thanks to the rapid development in polymeric biomaterial-based technologies to model and produce biomaterials, such as electrospinning, bio/3D printing, and nanofabrication, specific biomaterials with desired properties can be produced for immune modulation by regulating signalling cascades and cell-cell interactions that ultimately lead to immune modulation/engineering and the well-being of the individual (Figure 1).^5^ This work focuses on the current state of art, the attributes of the polymer-based biomaterials and their limitations in immune engineering applications. This review work not only elaborates on the advantages of polymeric biomaterials in immune engineering, but also critically analyses the promise of these biomaterials in immune engineering.

## Characteristics of polymeric biomaterials for immune engineering

**Figure 2.**
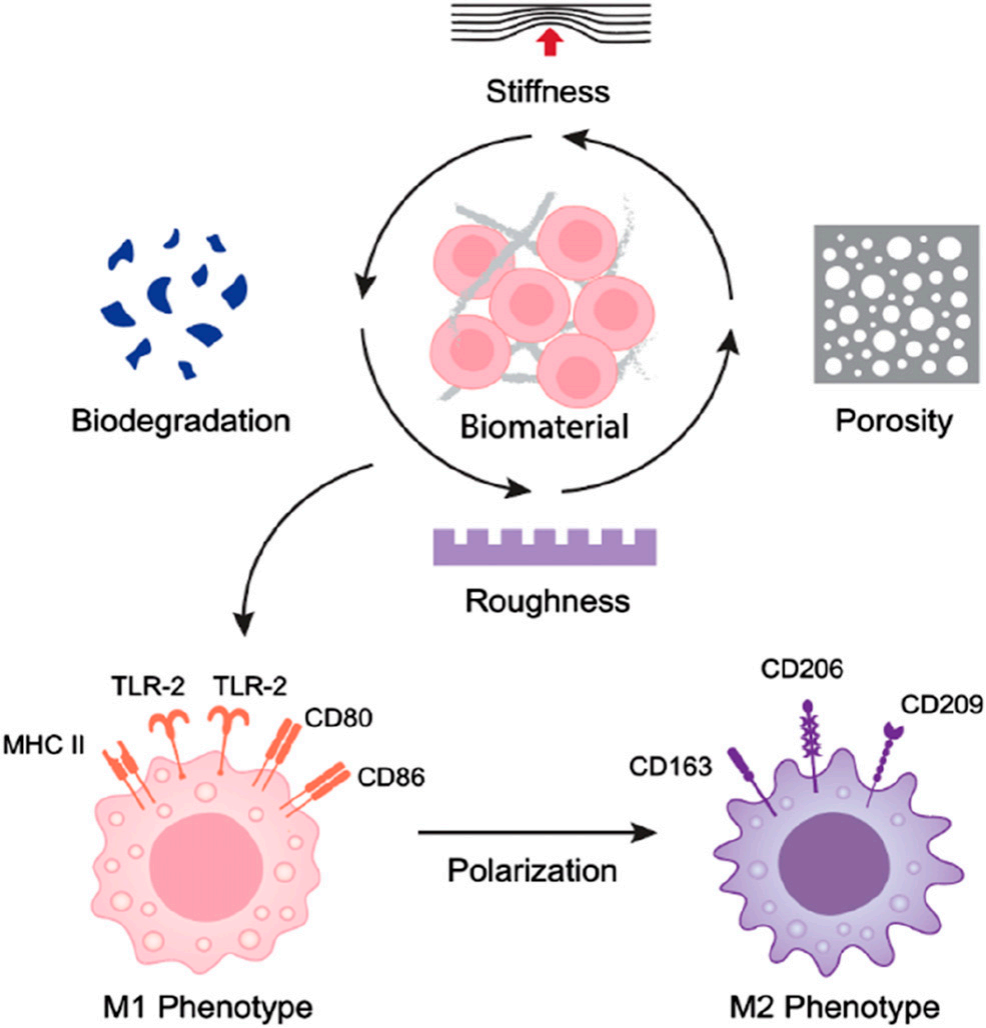
Property of the biomaterials for immune engineering applications. Different properties like stiffness, roughness, porosity, degradability has important role in immune cell activation and macrophage polarization for immune engineering.

The host organism responds to the applied biomaterial through a foreign body response (FBR). This response results from the significant biochemical and physiological interactions between the biomaterial and the host immune microenvironment.^5^ The functional performance of the immune-modulatory polymeric biomaterials depends on various structural and functional properties of the biomaterial, which are also influenced by the host immune microenvironment. The characteristics (Figure 2) of the biomaterials that are being used for immune engineering are discussed below:

(a) *Molecular weight of compositional polymer:* It has been studied that the molecular weight of the compositional polymers of a biomaterial has a strong influence on immunogenicity. The molecular weight of different compositional polymeric elements influences the antigen release profiles.^6^ Moreover, certain properties, such as adjuvant properties, have also been influenced by the molecular weight of compositional polymers. For example, the adjuvant-like property of poly-N-isopropyl acrylamide polymer (PNiPAAm) has been reported to be significant with its high molecular weight, which can lead to an elevated immune response.^7^ Another study reported that high molecular weight surface-grafted polyethylene glycol (PEG) significantly influences protein adsorption.^8^ Hence, the molecular weight of the compositional polymer plays an important role in facilitating the biomaterial’s functionality.
(b) *Particle size:* The particle size of the compositional elements of the polymeric biomaterial is an important criterion for its efficiency. It has been found that polymeric particles with a size of around 62-306 nm have a significant adjuvant-like effect.^9^ The high surface area provided by the nano-sized particles facilitates the adsorption of antigens. These nano-sized particles influence the immunological response through antigen uptake by antigen-presenting cells (APCs).^10^
(c) *Degree of crosslinking of compositional elements/polymers of the biomaterial:* Crosslinking of the polymer chains of the compositional polymers in the biomaterial is an important factor for exhibiting high efficiency. Crosslinking of polymers can be induced primarily through physical or chemical methods. It has been reported that crosslinking of collagen-based biomaterials significantly enhances the properties of the biomaterial, such as modulus and tensile strength.^11^ This ultimately influences the macrophage-specific inflammatory responses. A high degree of crosslinking facilitates macrophage activation.^12,13^
(d) *Hydrophilicity/Hydrophobicity of biomaterial:* The hydrophilic/hydrophobic properties of the biomaterial play a significant role in eliciting an immune response. High hydrophilicity generally does not support protein absorption. Moreover, biomaterials with high hydrophilic surfaces will stimulate a notable secretion of cytokines such as IL-1, IL-6, IL-8, and IL-10. Although the hydrophilic properties of natural polymers like alginate and hyaluronan often trigger immune responses, research has also demonstrated significant cell adhesion properties of hydrophilic biomaterials.^14,15^ On the other hand, the hydrophobicity of polymeric components of a biomaterial interferes with protein adsorption, ultimately resulting in an immunologically inert material. This, in turn, facilitates the biocompatibility of hydrophobic biomaterials.^14^ Moreover, hydrophobic characteristics also support other properties, such as the adjuvant-like properties of the biomaterial.^16^
(e) *Surface Topography and roughness:* Surface topography and roughness have a significant influence on immune response.^17^ Various studies have shown that changes in surface pattern, topography, and roughness influence macrophage response.^18^ It has been reported that surface-modified biomaterials with functional groups like hydroxyl (-OH) and amino (-NH2) groups also have a significant impact on the biomaterial’s ability to activate an immune response. The hydroxyl (-OH) group stimulates the immune response by activating different signaling pathways, such as IL-3, migration inhibitory factor (MIF)-Jun activation domain-binding protein 1 (MIF-JAB1), and oncostatin M signaling.^19^ On the other hand, biomaterial surfaces modified with amino (-NH2) groups reduce the immune response by blocking leukocyte adsorption.
(f) *Pore size:* Pore size of biomaterials also has an influence on macrophage behavior. It has been found that larger pore sizes facilitate high protein adsorption.^20^ Larger pore diameters ranging from 460 μm to 4000 μm facilitate the inflammatory response.
(g) *Biomaterial Architecture:* The architecture of the biomaterial plays an important role in immune cell activation. Biomaterials with a diameter of 4-5 µm have been found to be significant in immune cell activation. It has been observed that these micro-size particles provide the necessary contact area for T-cell activation.^21^
(h) *Degradability and rigidity of the biomaterial:* Degradability of the biomaterial is an important aspect to assess its effectiveness. It has been reported that the rate of degradation influences the rigidity of the biomaterial, which affects the host immune response at a significant level.^22^ Studies indicate that the host innate immunity responds to the degradation rate of biomaterials, such as decellularized extracellular matrix, which leads to the development of M2 macrophages from M1 macrophages.^23^ Rapid degradation of the biomaterial could negatively influence the regeneration process, as it causes a lack of adequate extracellular matrix (ECM) support for the cells, whereas slow degradation of the biomaterials could result in encapsulation of the biomaterial. In both cases, this can trigger an immune response.^24–27^ On the other hand, the rigidity of the biomaterial itself also influences immune cell activation and proliferation. It has been demonstrated that notable rigidity is associated with significant proliferation of CD4+ and CD8+ T cells in vitro (Table. 1).

**Table 1.** Cell response and rigidity of biomaterial for immune engineering applications.

Cells	Rigidity	Significance	Ref
CD4+ and CD8+ T cell	100-200 kPa	Enhanced T-Cell proliferation	^21,28,29^
B-Cells	20 kPa	Enhanced B-cell activation	^21,30^
Hematopoietic stem cells	200 kPa	Enhanced cell spreading	^21,31^

## Diversity of polymeric biomaterials and immune engineering

**Figure 3.**
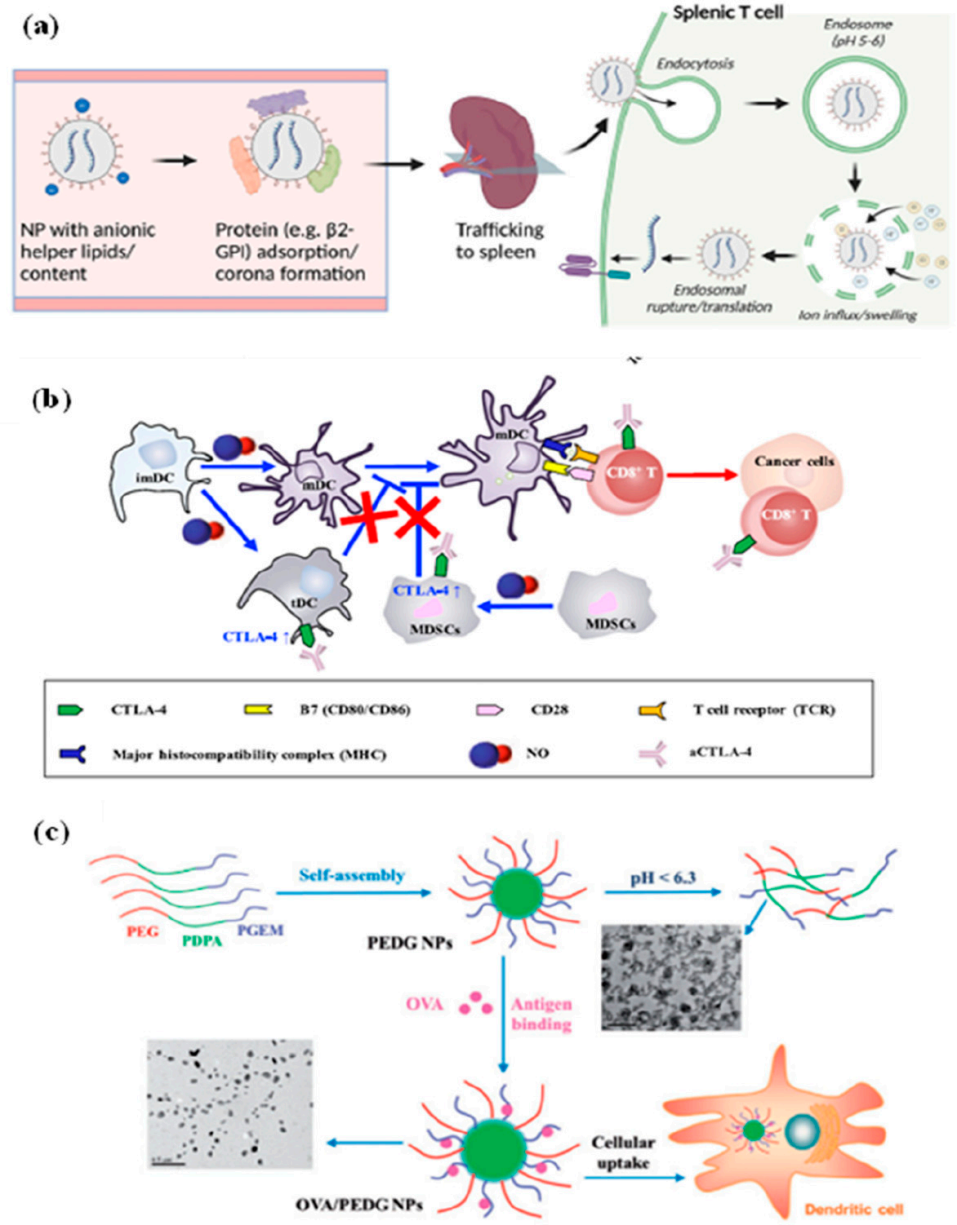
Application of polymeric material in immune modulation. (a) Polymer based gene delivery systems to immune cells in *vivo*. It majorly describes nanoparticles (NPs) splenic tropism for T cell transfection in *vivo*. (Represented after permission taken from Jain et al., 2024, Wiley, ^106^); (b) Gelatin and Pluronic® F127 based hydrogel for anti-tumour therapeutic application. The study demonstrates a nitric acid (NO) donor, S-nitrosoglutathione (GSNO) induce the expansion of natural killer cells. (Represented after permission taken from Kim et al., 2022, Nature, ^107^); (c) PEG-b-PDPA-b-PGEM copolymer self-assembled nanoparticles for protein antigen delivery to dendritic cells (Represented after permission taken from,^108^ Oxford Academy, Li et al., 2017).

Different polymeric systems have been reported that have been utilised for immune-engineering application (Figure 3) are discussed below:

### Natural polymers

Different natural polymers have been reported that have potentiality for immune engineering applications (Table.2). They are discussed as follows:

(a) *Collagen:* Collagen is one of the most common materials used for tissue engineering because of its abundance in skin, tendons, and bone. Its strength and structural integrity play a major role in immune engineering, serving as a substrate for immune cell adhesion of macrophages and proficient antigen-presenting cells, such as dendritic cells, as well as promoting wound healing and tissue repair. Collagen facilitates the M1 to M2 polarization, which is characterized by the production of anti-inflammatory cytokines such as TGF-β and IL-10.^32^ Its degradation is mediated by enzymes such as collagenases, as well as by oxidation in the case of oxidative stress, within a period of 2-4 weeks.^33^
(b) *Hyaluronic Acid (HA):* Hyaluronic acid is another material that is highly accessible due to its high concentrations in connective tissue. Its molecular structure, as a glycosaminoglycan, serves as a conductive environment for cell migration, cell proliferation, and regulation of pro-inflammatory processes. HA is the main component of hydrogels, serving as a scaffold for the regulation of immune response by T cell activation, resulting in IL-10 and TGF-β.^34^ HA degradation is primarily regulated by the enzyme hyaluronidase within a timeframe of 2 to 6 weeks, depending on the concentration.^35^
(c) *Chitosan:* Chitosan is derived from chitin, which is the polymer composing the exoskeleton of arthropods. As a molecule, it is amphiphilic, but its characteristics are pH-dependent due to the presence of amine and hydroxyl groups, making it more hydrophilic at lower pH levels. Chitosan is considered non-toxic and has great potential for drug delivery systems for immune therapies, as well as for interaction with macrophages, resulting in immune modulation and wound healing processes. It can also be used as an adjuvant in vaccines to initiate an immune response to selected pathogens. The degradation period varies and is regulated by the degree of deacetylation. Chitosan can interact with macrophages and lead to the production of anti-inflammatory IL-10 and IL-4, which result from M2 polarization.^36^
(d) *Alginates:* Extracted mainly from the class Phaeophyceae, brown seaweeds contain these polysaccharides. Their natural ability to form hydrogels makes them highly suitable for delivery systems and cell encapsulation. Their degradation is primarily regulated by alginate lyases over a period of 2 to 4 weeks after being introduced in vivo.^37^ The immune modulation of alginates is related to a reduction in inflammation by promoting M2 polarization and the production of anti-inflammatory cytokines, such as IL-10.^38^
(e) *Fibrin:* A natural protein has its main function in wound healing processes by initiating blood clot formation together with factor XIII and platelets. It is degraded by proteases such as plasmin for up to 3 weeks after formation.^39^ Its long fibers can promote the adhesion of macrophages that are polarized into the M2 phenotype, with the secretion of anti-inflammatory cell responses.^40^

**Table 2.** Natural polymeric biomaterials and their role in immune engineering^14,41^.

Natural polymer	Role in immune engineering		
	Receptor	Signalling pathway	Activating cells
Collagen	Discoidin domain receptor (DDR), osteoclast-associated receptor (OSCAR), and integrin receptors.	protein kinases (MAPK), ERK, NFkB and cytokines.	(OSCAR)-Ca++
			Pathways facilitate cytokine release
Gelatin	Integrin (avb3 and avb5)	FAK, ErK, and Ras.	High level of IL-10, Arginase specific for M-2 macrophage
Fibrin	Mac-1 receptor	Mac-1 mediated signalling	High production in IL-8, IL-6, TNF-a, and reactive oxygen species in leukocytes
Chitosan	Dectin-1 and TLR	Syk, CARD90, MAPK	IL-2, CXCL-2 and 1

### Synthetic polymers

Various synthetic polymers have also been found efficient in immune engineering (Table.3) are discussed below:

(a) *Polylactic acid (PLA):* The main source of this polymer is lactic acid molecules that can be obtained from renewable sources like corn starch. Its main application in tissue engineering is for scaffold creation with flexible mechanical properties and in delivery systems, such as wound healing promotion or a matrix for immune cells. The degradation rate varies, with the main mechanism being hydrolysis; the rate depends on pH and the microenvironment. Inflammatory conditions promote the PLA degradation rate.^42^ Scaffolds and delivery systems made from PLA promote wound healing by M2 phenotype polarization and the release of anti-inflammatory cytokines like IL-10.^43^
(b) *Polyglycolic acid (PGA):* A synthetic polymer composed of glycolic acid is rich in ester bonds and can degrade by hydrolysis, which can take up to a few months.^44,45^ The main mechanism utilised in immune tissue engineering is M2 macrophage phenotype polarization, accompanied by the release of IL-10 and TGF-β anti-inflammatory cytokines, thus promoting the wound healing process and tissue repair while downregulating chronic local inflammation.^46^
(c) *Polylactic-co-glycolic acid (PLGA):* A copolymer composed of both lactic acid and glycolic acid has properties that depend on the corresponding concentration ratios of both materials, which can modulate controlled release as well as encapsulation potential.^47^ The main mechanism of tissue repair and immune modulation is through the secretion of anti-inflammatory cytokines by M2 phenotype polarization of macrophages, along with providing support for immune cell growth.^46^
(d) *Polyethylene glycol (PEG):* A synthetic polymer with significant implications and a tremendous impact in recent years on biomedical sciences, particularly in immunology and tissue engineering, is PEG. It is considered a gold standard material for biomedical applications.^48^ The molecule is highly hydrophilic; therefore, it is water-soluble, making it an excellent material for drug delivery systems. PEG can also be designed to functionalize active groups to modify its function in relation to the microenvironment, resulting in tissue regeneration and wound healing through controlled release mechanisms.^49^ The mechanisms of M2 phenotype polarization are contributed to by.^50^
(e) *Polycaprolactone (PCL):* Polycaprolactone (PCL) is a synthetic polymer that has emerged as a versatile material due to its unique combination of mechanical properties and slow degradation rate, making it particularly useful for long-term implantable devices and scaffolds. As highlighted by,^51^ PCL’s excellent malleability allows for the tailoring of various forms, such as fibers, films, and microspheres, which are ideal for controlled drug release and tissue regeneration applications. The polymer’s high degree of crystallinity and low melting point also make it suitable for 3D printing technologies in the production of scaffolds for tissue engineering.^52^ Furthermore, PCL’s ability to be functionalized with bioactive molecules or incorporated with other biomaterials enables the modulation of its properties, which can be utilised to enhance cell growth and tissue formation.^53^ Notably, PCL has shown promise in promoting the regeneration of bone, cartilage, and nerve tissues through both physical support and the controlled release of therapeutic agents.^54^

**Table 3.** Synthetic biomaterials and their role in immunomodulation.

Synthetic polymers	Role in immune engineering			
	Receptor	Signalling pathway	Activating cells	Refs
PLA	TLR2/TLR4	TLR2/4 → MyD88 → NF-κB, MAPK	Dendritic cells, macrophages	[55]
PGA	Integrins (mostly α5 family)	Integrin → FAK → PI3K/Akt	T cells, macrophages	[56]
PLGA	TLR4, CD80/CD86	TLR4 → NF-κB; CD80/86 → CD28 → PI3K → Akt → mTOR	Dendritic cells, T cells	[57]
PEG	CD44, IL-2R	CD44 → Rho GTPases; IL-2R → JAK/STAT → STAT5	Neutrophiles, macrophages, dendritic cells	[58]
PCL	Integrins, TLR2	Integrin → Src/FAK → ERK; TLR2 → NF-κB	Macrophages, dendritic cells	[59]

Different natural and synthetic polymer-based biomaterial have the commercial presence focuses on the immune engineering in the process of tissue regeneration. The diversity of their property (eg. degradation time), product cost and target organ specific information are discussed in Table.4. Significant applications of various polymeric material in immune modulation and bioengineering described in different research reports are represented in Figure 3.

**Table 4.** Commercial and other reported biomaterial focuses on immune engineering.

Biomaterial		Trade name or applied name	Approximate product cost	Degradation time (week)	Target organ	Ref
Natural polymer	Collagen	-Integra ® (bilayer scaffold)	$538-$12,613	2.0-16.0	Nerve, cartilage, skin, cornea	^60–69^
		-Chondro-guide ® (membrane)				
		-NeuraGen® (conduit)				
		-BioCornea® (lamellar sheet)				
	Gelatin	GelMA (Gelatin methacrylate hydrogel)	$110,00-$560,00	1.0-16.0	Lymph nodes, spleen, thymus, bone marrow, skin	^70–73^
	Fibrin	EVICEL® (hydrogel)	$93.95-$700	∼2.2	Skin and mucosal tissues	^70,74–77^
		TISSEEL®(hydrogel)				
		Artiss® (hydrogel)				
	Chitosan	KytoCel® (hydrogel)	$12.90-$424.90	∼2.2-20	Skin, bone, cartilage, nerve, liver, muscle	^78–82^
		Chitosan® (hydrogel)				
Synthetic polymer	PEG	3-D Life® Dextran-PEG/PVA-PEG Hydrogels (Cellendes) (hydrogel)	$188.27	∼4.0-8.5	Lymph nodes, intestinal epithelium, skin, bone	^83–86^
	PLA	RESOMER® (powder)	$858.00	∼30.0	Spleen, lymph nodes	^60,87–89^
	PLLA	PLLA Gelacell™ (porous scaffold)	$50-$400	∼30.0	Spleen, lymph nodes	^90,91^
	PGA	NEUROTUBE device (conduit)	$1200-$3500	∼18.0	Thymus, lymph nodes, and spleen	^92–94^
	PLGA	Ethisorb (mesh) lipid@PLGA hNPs (nanoparticles)	$100-$5500	∼3.0	Bone tissue, liver, periodontal tissues, lymph nodes	^47,95–99^
		Trelstar (microspheres)				
		Resomer® RG 504 H, Poly(D,L-lactide-co-glycolide) (powder)				
	PCL	NEUROLAC® (conduit)* Clinical trial or limited-release stage	$1500-$30000	∼13.0	Cartilage, vascular, skin, nerve	^100–105^

## Polymeric biomaterial, macrophage polarization and immune engineering

**Figure 4.**
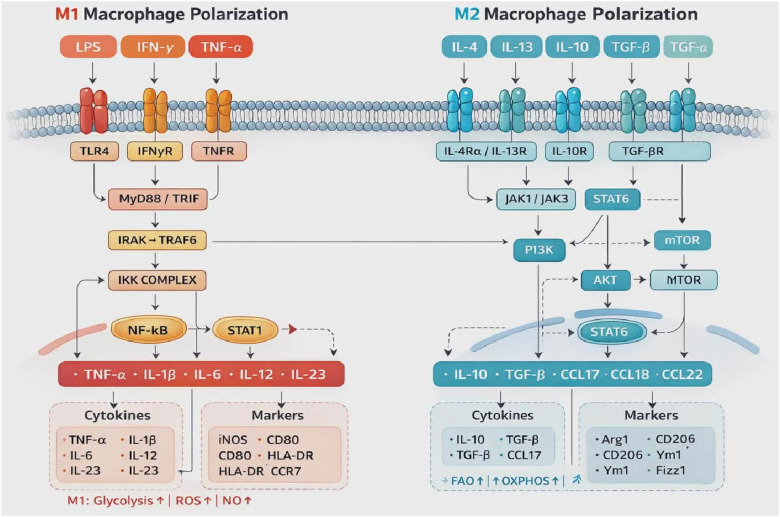
Classical cytokine-driven signaling pathways and functions of M1 and M2 macrophages for immune modulation. M1 macrophages are activated by inflammatory signals whereas M2 macrophages are activated by anti-inflammatory cytokines. They produce tissue-repair mediators such as IL-10, TGF-β, and chemokines (CCL17, CCL18, CCL22) for immune modulation and engineering^117^.

Macrophage phenotype exhibits remarkable plasticity and responsiveness to various environmental cues, including those presented by polymeric biomaterials. These biomaterials facilitate the transition of M1 to M2 macrophages (i.e., macrophage polarization), and this transition from an M1 to an M2 macrophage phenotype is governed by a complex interaction of physicochemical and biochemical signals^109^ (Figure 4.). The shift to the M2 phenotype occurs when inflammatory signals subside, and anti-inflammatory cytokines such as IL-4, IL-13, IL-10, or components released during the pro-apoptotic cascade (as shown in Table 5) become prevalent.^110,111^ Designed biomaterials can function as immune-modulatory platforms, actively influencing this phenotypic switch by interacting with specific cell surface receptors and triggering subsequent intracellular signalling cascades, which ultimately lead to macrophage polarization and phenotype switching.^112^ Numerous stimuli are associated with the molecular pathways that drive macrophage polarization. In the M1 state, macrophages are activated by pathogen-associated molecular patterns (PAMPs) or damage-associated molecular patterns (DAMPs), recognized by receptors such as Toll-like receptors (TLR2, TLR4), Dectin-1, or integrin complexes like Mac-1. These receptor-ligand interactions activate specific signalling pathways, such as the MyD88-dependent pathway, which triggers downstream signalling leading to the expression of pro-inflammatory cytokines such as IL-1β, IL-6, TNF-α, and IL-12. Specific transcription factors like STAT1 and IRF5, which are involved in this mechanism, further enhance the M1 transcriptional program, reinforcing the classical activation phenotype.^113–115^ This variety of intracellular signal transduction phenomenon and activation of macrophage is facilitated significantly by different polymeric biomaterials.^115,116^

**Table 5.** Role of macrophages in immune regulation^114,115^.

Macrophage	Transcription factors	Cytokines	Chemokines	Functions
M1	NF-κB, STAT1, IRF1, IRF5, HIF-1α, KLF6	NO, TNF-α, IL-1β, IL-6, IL-12, IL-23	CXCL9, CXCL10, CXCL11	Pro-inflammatory, microbicidal activity, clearance of pathogen
M2	STAT3, STAT6, IRF4, KLF4, PPARγ, cMaf, cMyc	IL-10, TGF-β	CCL17, CCL18, CCL22	Anti-inflammatory, immune regulators, tissue repair

## Advantages of scaffold/matrix based immune-engineering methods

Polymeric biomaterials were designed with specific pore sizes, surface topographies, and mechanical properties to influence cellular behaviour and immune modulation.^118^ Due to their important properties like biocompatibility exhibited several advantages for immune engineering applications,^119^ which are discussed below: (a) *Modulating Macrophage Polarization:* Polymeric biomaterials significantly influence macrophage polarization by promoting the transition from pro-inflammatory M1 macrophages to anti-inflammatory and tissue-reparative M2 macrophages. This mechanism is critical for reducing chronic inflammation and encouraging tissue engineering.^5,120^ Through their biophysical properties, strategically designed polymer scaffolds are capable of directing macrophage responses by modulating mechanical cues, surface topography, and degradation kinetics, thereby enabling precise tuning of the local immune microenvironment.^121^ The interactions between biomaterials and the immune microenvironment are mediated through the controlled and spatially localized delivery of bioactive substances, including proteins and nucleic acids.^5^ Furthermore, the functionalization of polymeric biomaterials’ surfaces with bioactive molecules, such as cytokines or growth factors, enables precise modulation of immune cell behaviour, enhancing the reparative capacity of the tissue.^122,123^ (b) *Structural and Functional Support*: Polymeric biomaterials like collagen, chitosan facilitate immune engineering through balancing mechanical integrity of the biomaterial and its bioactivity.^32,36,124^ (c) *Targeted Delivery of Bioactive Molecules*: Engineered biomaterials act as carriers that enable the controlled and localized release of immunologically active agents, such as cytokines, growth factors, and nucleic acids. These systems provide an instructive microenvironment for immune and stromal cells by mimicking the biochemical cues of the native extracellular matrix.^125,126^ The incorporation of nanoparticles into biomaterials can further enhance targeted delivery, ensuring that therapeutic interventions are precisely focused and that systemic side effects are minimized.^123,127^ Furthermore, immune-modulating biomaterials can be designed to present antigens in an immunologically relevant context to enhance adaptive immune responses. Ultimately, these multifunctional platforms not only modulate cellular and immune responses but also serve as dynamic environments to orchestrate regeneration in a spatially and temporally controlled manner^125^; (d) *Effective therapeutic option with reduced systemic side effects*: Innovative biomaterial designs, such as nanoparticle-based polymeric systems and growth factor-loaded biomaterials,^125,128^ utilise advanced materials to enhance compatibility with host tissues, thereby reducing the risk of adverse immune reactions. Researchers emphasize the importance of engineering scaffolds to minimize reliance on systemic immunosuppressants and instead optimize localized interactions that encourage healing and reduce immune interference.^127,128^ (e) *Could be used with minimum invasive procedure*: Polymeric biomaterials in injectable liquid form, which can undergo phase changes in response to physiological conditions (pH, temperature), provide an additional advantage due to their minimally invasive administration and high efficiency.^129^ The injectable polymeric systems offer the distinct advantage of in situ gelation at the target site, enabling minimally invasive administration and localized, sustained release of therapeutic agents. This approach significantly reduces systemic toxicity and enhances patient compliance, particularly in long-term treatment settings.^129,130^ These materials are exceptionally well-suited for diverse biomedical applications, including cancer therapy, musculoskeletal tissue repair, and neural regeneration.^129,130^

## Challenges and prospects of polymer-based biomaterials for immune engineering

The bioengineered polymeric immunomodulatory biomaterials have been developed to modulate the foreign body response (FBR). To achieve specific immunomodulatory effects and regulate the FBR, these biomaterials are designed to interact with the immune microenvironment via the involvement of immune-related substances or cells.^5,18^ Hence, the integration of immunological principles into biomaterial design is a pivotal strategy in advancing next-generation biomaterials for immune engineering applications.

The application of polymeric biomaterials in immune engineering faces several significant challenges: (a) *Undesirable pro-inflammatory responses from polymeric biomaterial* One of the primary concerns in immune engineering is the potential for biomaterials to provoke chronic inflammation, fibrosis, or immune rejection. The strong inflammatory response can be detrimental to the success of tissue engineering materials by mediating the development of chronic inflammation. This process subsequently leads to host rejection or fibrosis of implanted grafts and medical devices.^131,132^ Many polymeric materials can trigger an excessive immune reaction, complicating long-term integration within the host tissue. Persistent activation of dendritic cells and M1-type macrophages at the site of implantation leads to a sustained release of pro-inflammatory cytokines such as IL-1β, TNF-α, and IL-6, which further compromise healing and regeneration.^131^ Strategies such as surface modification, anti-inflammatory coatings, and optimized polymer degradation rates are crucial to mitigating these risks.^5^ *(b) Unwanted cytotoxic effect and markers:* Endotoxin-like effects of certain polymers, such as marine-derived collagen and gelatin extracts,^133^ can hinder the overall efficiency of the polymeric biomaterial and facilitate the possibility of unwanted toxicity in the host body. Biomaterials such as chitosan, although often perceived as biologically inert, can exhibit highly complex and at times contradictory interactions with the immune system. These interactions are profoundly influenced by factors such as chemical modifications, degree of purity, and the specific methodologies employed during polymer synthesis and processing. Even subtle variations in these parameters can markedly alter the immunomodulatory profile of chitosan, potentially shifting its effects from immunosuppressive to pro-inflammatory. Consequently, while chitosan-based materials may offer therapeutic benefits in tissue repair and regeneration, they also carry the risk of inducing unintended inflammatory or cytotoxic responses if not rigorously controlled.^133^ Further supporting this, endotoxin contamination represents one of the critical challenges in the development of biomaterials, as it can profoundly impair both the functional performance of the biomaterial and the success of engineered tissues. Endotoxins, predominantly lipopolysaccharides (LPS) derived from the outer membrane of Gram-negative bacteria, are commonly present in naturally sourced biomaterials due to their biological origin and the inherent difficulties in achieving complete sterility. However, synthetic biomaterials are also susceptible, as endotoxin contamination may arise through contact with contaminated reagents, equipment, or airborne particles during processing, storage, or handling.^131^ These findings underscore the imperative for stringent quality control measures and standardization to ensure the safe, predictable, and immune-compatible application of polymeric biomaterials. Therefore, endotoxin quantification (e.g., LAL assay) should be considered a baseline release/quality marker when interpreting peri-implant inflammatory outcomes.^134^ As the markers of the immune response, the clinically relevant cellular immune readouts can be captured through routine hematology and targeted immune markers, including CBC with differential (total leukocytes, neutrophils; NLR when informative), and eosinophils with serum IgE when a hypersensitivity component is suspected (e.g., collagen/gelatin/chitosan). Moreover, complement specific markers (C3/C4 and/or total complement activity, CH50) may support identification of complement-mediated intolerance, in specialized settings. Furthermore, inflammatory cytokines such as IL-6 (serum or local fluid) may serve as an early indicator of excessive inflammatory activation.^135^ Furthermore, considering the foreign-body inflammation from infection is another important aspect. This includes serial CRP/ESR trends (persistence without infection suggests sterile chronic inflammation), and microbiological culture of wound exudate.^136^ (c) *Unpredictability of host immune system and microenvironment:* The immune response varies significantly across individuals due to genetic diversity and tissue-specific microenvironments. Standardized predictive models for biocompatibility assessment remain a significant limitation in translating in vitro findings to reliable in vivo applications.^137,138^ Recent studies emphasize the need for computational modelling and advanced immunological profiling techniques to better predict host responses.^122,139^ Ultimately, bridging the gap between in vitro prediction and in vivo performance will require not only technological innovations but also a conceptual shift toward personalized biomaterials, where patient-specific immune data guide material selection and functionalization. This integrative approach holds the potential to reduce immune complications and increase the therapeutic success of biomaterial-based interventions; (d) *Balancing biomaterial properties and bioactivity*: Biomaterial design requires a delicate balance between structural integrity and immunological activity. Mechanically robust materials may induce an inflammatory response, whereas highly immunologically inert materials often lack sufficient bioactivity to promote tissue regeneration.^119,132^ Recent advancements in biomaterial synthesis focus on hybrid polymers with tunable properties that optimize both mechanical stability and immune responsiveness^140^; *(e) challenges related to computational modelling and predictive modelling for biomaterial design:* Previous research reports that predictive computation based modelling could be an exciting approach for predicting immune engineering based inflammatory responses.^141^ It is highly useful to utilise modelling based approaches to predict the efficiency of an immune engineering specific biomaterial; however, this method faces a significant challenge related to selection of proper physic based or mathematics based models due to robust data sets considering dynamic physiological environmental parameters^142^; (*f*) *Ethical Clinical challenges and ethical and regulatory constraints*: The clinical translation of polymeric biomaterials faces ethical challenges related to preclinical testing in animal and human models. Natural polymers face significant barriers to clinical implementation under FDA and EU MDR (Medical Device Regulation 2017/745) regulations, including batch-to-batch variability in quality, immunological unpredictability, and limitations in scalable manufacturing. In contrast, synthetic polymers dominate clinical applications due to their high reproducibility, regulatory compatibility, and well-characterised degradation profiles.^143^ Hybrid polymer systems offer a promising intermediate approach; however, their successful clinical translation depends on overcoming tissue-material crosstalk complexity, degradation mismatches, and challenges associated with production.^144^ Finally, clinical practice prioritizes predictability, procedural robustness, and risk management over maximal biological efficacy when the outcomes remain uncertain ^145^. Regulatory approvals require extensive biocompatibility assessments to ensure safety while maintaining innovation in biomaterial applications.^146^ The ethical principles for human clinical translation, as described in the 1979 Belmont Report, are threefold: respect for persons, beneficence, and justice.^145–147^ Thus, while ethical and regulatory frameworks are indispensable for safeguarding patients, they must also evolve in parallel with scientific progress;

Despite facing challenges, polymer-based immune engineering biomaterials could provide significant advancements, particularly in: *(1) Automation-mediated immune engineering:* Designing polymeric devices through machine learning-based applications could provide an edge over trial-and-error synthetic methods. This approach will not only be cost-effective but also offer the opportunity to explore different possibilities for designing biomaterials for immune engineering^147^; *(2) Personalized Immunotherapy:* Precision medicine approaches involve tailoring biomaterial properties to individual patients’ genetic and immunological profiles. By following this strategy, polymeric biomaterials could allow for customized immune modulation, further improving therapeutic efficacy while minimizing adverse effects^127^; *(3) New generation of Engineered Living Materials (ELMs):* Novel ELMs can be developed through the utilisation of advanced methods like 4D bioprinting and volumetric bioprinting.^148^ These materials leverage the autonomous characteristics of living organisms to enhance responsiveness and therapeutic control^127,149^; *(4) Nanoparticle carriers and implantable depots for immune cell targets:* Drug-loaded implantable polymeric depots can be developed using a combination of natural and synthetic polymers for controlled-release therapies that adapt to immune system fluctuations; *(5) Delivery of stem cell-derived extracellular vesicles (EVs):* In recent times, mesenchymal stem cell-derived extracellular vesicles or microsomes have shown promise in tissue engineering applications.^150^ Polymeric biomaterials could be utilised as vehicles for EVs, thereby facilitating immune engineering; *(6) Piezoelectric biomaterials for macrophage polarization:* Research has reported that polymers with piezoelectric properties, like poly(vinylidene fluoride), have the ability to influence macrophage polarization (especially M1).^151^ Hence, biomaterials with piezoelectric properties could be an exciting avenue for further research to design advanced immune engineering materials; *(7) Biosensing and Diagnostic Applications:* Immune-responsive biomaterials hold promise for developing advanced biosensors and diagnostic tools. These materials enable early detection and real-time monitoring of immune-related disorders, improving personalized treatment strategies.^127^

## Conclusion

Polymeric biomaterials have transitioned from inert components to active immunomodulators, fundamentally reshaping the environment of immune tissue engineering. Their effectiveness hinges on a nuanced interplay of physicochemical properties, including molecular weight, particle size, hydrophilicity, surface topography, pore size, architecture, and degradability. The understanding that these properties exert their influence across multiple length scales, from molecular interactions to bulk biophysical characteristics, highlights the need for a holistic design approach. This multi-scale interdependence dictates that optimizing a single property in isolation is insufficient; rather, synergistic interactions must be considered to achieve the desired immune outcomes. Furthermore, the recognition that immune cells are mechanosensitive and that their behaviour is context-dependent underscores a critical translational challenge, emphasizing the need for tailored solutions and more physiologically relevant in vitro models. Both natural and synthetic polymers offer unique advantages. Natural polymers provide inherent biocompatibility, biodegradability, and extracellular matrix (ECM) mimicry, while synthetic polymers offer unparalleled tunability, mechanical stability, and precise control over degradation and therapeutic delivery. The strategic imperative for future advancements lies in the development of hybrid materials that synergistically combine the bioactivity of natural polymers with the engineered precision of synthetic counterparts. The core mechanism of immune modulation by these materials often revolves around guiding macrophage polarization from pro-inflammatory M1 to pro-regenerative M2 phenotypes. This process is intimately linked to mitochondrial function, where biomaterials can manipulate cellular metabolism, reactive oxygen species levels, and ion homeostasis to steer macrophage fate. Beyond cellular reprogramming, polymeric biomaterials excel in the controlled and localized delivery of bioactive molecules, enhancing therapeutic efficacy and minimizing systemic side effects across drug, gene, and vaccine delivery platforms. Despite these profound advancements, significant challenges persist. Managing adverse immune reactions, particularly the foreign body reaction, remains a critical hurdle. Balancing material properties for optimal function with immunological inertness and navigating the inherent unpredictability of host immune responses continue to demand rigorous research. Furthermore, the complexities of clinical translation—including manufacturing scalability, ensuring consistent product efficacy, and overcoming immune evasion mechanisms in diseases like cancer—require innovative solutions. Realizing the full potential of polymeric biomaterials in immune tissue engineering necessitates continued and robust interdisciplinary collaboration. The convergence of materials science, immunology, biomedical engineering, and synthetic biology is essential to unravel the complex material-immune interactions, overcome existing limitations, and translate these groundbreaking scientific advancements into clinically impactful therapies and diagnostic tools.
